# The role and mechanisms of microbes in dichlorodiphenyltrichloroethane (DDT) and its residues bioremediation

**DOI:** 10.1016/j.btre.2024.e00835

**Published:** 2024-03-11

**Authors:** Girma Ebsa, Birhanu Gizaw, Mesele Admassie, Tizazu Degu, Tesfaye Alemu

**Affiliations:** a Department of Microbial, Cellular and Molecular Biology, Addis Ababa University, P. O. Box: 1176, Addis Ababa, Ethiopia; b Department of Crop Protection, Ethiopian Institute of Agricultural Research, P. O. Box: 2003, Addis Ababa, Ethiopia

**Keywords:** Dichlorodiphenyldichloroethane, Dichlorodiphenyldichloroethylene, Dichlorodiphenyltrichloroethane, Microbial remediation, Soil, Water

## Abstract

Environmental contamination with dichlorodiphenyltrichloroethane (DDT) has sever effects on the ecosystem worldwide. DDT is a recalcitrant synthetic chemical with high toxicity and lipophilicity. It is also bioaccumulated in the food chain and causes genotoxic, estrogenic, carcinogenic, and mutagenic effects on aquatic organisms and humans. Microbial remediation mechanism and its enzymes are very important for removing DDT from environment. DDT and its main residues dichlorodiphenyldichloroethylene (DDE) and dichlorodiphenyldichloroethane (DDD) can biodegrade slowly in soil and water. To enhance this process, a number of strategies are proposed, such as bio-attenuation, biostimulation, bioaugmentation and the manipulation of environmental conditions to enhance the activity of microbial enzymes. The addition of organic matter and flooding of the soil enhance DDT degradation. Microbial candidates for DDT remediation include micro-algae, fungi and bacteria. This review provide brief information and recommendation on microbial DDT remediation and its mechanisms.

## Highlights

• Currently, a biological approach has been used as an environmentally-friendly and cost-effective treatment.
• Microbial remediation technology and microbial enzymes were used to degrade DDT.
• The abilities of microbes to degrade DDT are explained.
• The mechanisms, pathways of degradation, and application in contaminated soil and water is described.
• The review focuses on the most effective source for microbial DDT remediation and future perspective.

## Graphical abstract

**Figure:**
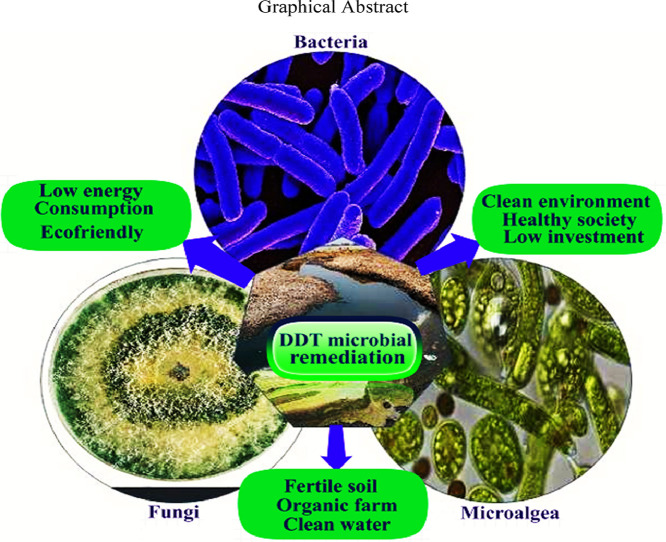

## 1 Introduction

The main drivers of environmental contamination include modern farming methods, industrialization, fast urbanization, and other human anthropogenic activities [1]. These activities release a significant amount of toxic pollutants into the environment and affect both animals and plants in the corresponding ecosystems [[2], [3], [4], [5], [6]]. It was the first synthetic insecticide chemical used all over the world. It has been used as an insecticide to combat pests in agriculture and diseases like malaria and typhus [[7], [8], [9]]. Recently, most nations banned the use of DDT because of its harmful effects on animals and human health through the food chain. DDT may lead to a variety of acute and chronic diseases in humans [10,11].

According to many research reports, less than 1 % of all DDT pesticides used are utilized to target pests; the remaining DDT pesticides are precipitated in the nearby water and soil. The remaining DDT in the ecosystem has a negative impact on the ecology [12,9]. DDT and its residuals have been found in both industrialized and developing nations' environments [13,14]. Due to its toxicity and persistence in the environment, environmentalist and health administrators, as well as members of the general public, have voiced concerns [15], [16], [17]. The breakdown of DDT insecticides frequently produces both toxic and non-toxic intermediates, which should be taken into consideration when developing a remediation strategy. Microbes, such as fungi, bacteria, microalgae, and others, are reported for DDT breakdown and used as bioweapons to fight DDT chemicals and caught the researcher's attention in recent years [18,19]. DDT degradation is dependent on natural reactions in the environment, such as chemical, biological, and physical. These reactions take place when a DDT pesticide particle interacts with a soil particle, a water molecule, or a living organism [20], [21], [22]. Abiotic and biotic components, as well as the physical and chemical characteristics of the soil, all have an impact on the rates of DDT degradation in soil and water [23,22]. In DDT degradation process, the presence of soil and water microbes is a very important factor, which depends on the environmental conditions of the soil and water [24,25].

DDT can be eliminated from soil and water using a variety of microbial remediation technologies [23,26]. Bacterial remediation of DDT in contaminated water and soil is based on the capability of bacterial cells to tolerate and accumulate DDT pollutants. The bacterial population and DDT content determine the rate of degradation [27,28]. Both aerobic and anaerobic conditions allow bacteria to degrade DDT. Some reported bacterial strains with degradation potential are Escherichia coli, Enterobacter aerogens, Enterobacter cloacae, Klebsiella pneumonia, Pseudomonas aeruginosa, Pseudomonas putida, Bacillus species, Hydrogenomonas, etc. Sulfate-reducing bacteria such as Desulfovibrio, Desulfotomaculum, Desulfobacter, and Desulfococcus genera are among the anaerobic bacteria found in sewage treatment systems [29,30].

Fungi remove DDT-pollutants by increasing their bioavailability and transforming them into less toxic forms [27,31]. Fungi are easy to grow and produce an adequate amount of biomass for DDT remediation [32]. Certain fungal strains have shown the ability to degrade DDT [33]. Fungi are a great option for DDT-cleanup of contaminated soil and water because of two crucial characteristics. Their secretion of many intra- and extracellular enzymes and the production of hyphal mesh of fungi. It protects the internal, sensitive organelles from the ill effects of DDT contaminants, which is an advantage of using fungal culture in water and soil treatment over bacterial culture [34,35]. The most studied fungi that have been found to be capable of degrading DDT, including *Saccharomyces cervisiae, Phanerochaete chrysosporium*, and *Trichoderma viridae* [36,37]. According to Sharma et al. [30], fungi convert DDT contaminants into important biochemicals and other valuable compounds industrially.

In addition to bacteria and fungi, algae also used for DDT remediation [[38], [39], [40]]. Phycoremediation is the use of algal species for biological DDT-contaminated water remediation [41]. Microalgae remove DDT pollutants through two processes: microalgae DDT accumulation and microalgae DDT degradation. A mechanism known as microalgae DDT accumulation allows microalgae to absorb and store DDT inside their cells, whereas microalgae DDT degradation is the natural way in which DDTs are broken down by microalgae into simpler molecules like carbon dioxide and water [42], [43], [44]. Microalgae and cyanobacteria have a potential application in the field of environmental pollutant remediation and are considered capable of degrading DDT. A large number of enzymes from microalgae have been found to be involved in DDT degradation [34]. Microalga S. obliquus and Green alga Chlorella fusca var. *vacuolata* have the capacity to degrade DDT [44], [45], [46].

For improving the protection of soil, environment, and human health in the future, setting legislation threshold limits and determining maximum residue limits for DDT pesticides is a key solution [47,48]. Soil contamination on the basis of DDT concentration is classified as: (i) negligible contamination (<50 μg kg^−1^ DDT), (ii) low contamination (50–500 μg kg^−1^), (iii) medium contamination (500–1000 μg kg^−1^), and (iv) high contamination (>1000 μg kg^−1^). Because DDT pesticides are persistent xenobiotics, they can have transboundary impacts. Therefore, legislation is required, both regionally and globally [25,48]. The aim of the current review paper is to discuss the role, mechanisms, and challenges linked to the microbial remediation of DDT and its metabolites.

## 2 Microbial DDT remediation

The process of converting DDT into low-molecular-weight chemicals by means of effective microorganism enzymes is known as microbial degradation of DDT pesticide [49], [50], [51]. Microbial DDT remediation is an economically viable and efficient method of degrading DDT that is typically reliant on the types of microorganisms and environmental conditions [49], [50], [51], [52]. It is a safe and eco-friendly technique for soil and water DDT contamination remedies. DDT is an extremely toxic chemical substance and can affect growth, reproduction, behavior, enzymes, and the DNA of microbes [12,53]. Therefore, eradicating this chemical from the environment is very essential. Microorganisms have enormous catabolic potential for the remediation of DDT pollutants and then breaking them down into less toxic compounds. Bacteria and fungi are a potential means of remediating DDT by secreting exudates as an energy source and cooperating together in the remediation process [52,[54], [55], [56]]. Saprophytic fungi can respond and develop resistance, metabolizing a wide variety of organic pollutants over time [57]. Many microbial species can play a role in DDT degradation (Table 1). White rot fungi species were able to degrade seventy (70) and thirty (30) percent of DDT, respectively, within twenty-one (21) days of incubation in a low-nitrogen medium [57,58]. Microbial DDT degradation" primarily refers to three processes: In the first, the original parent pesticide chemical is converted into soluble and non-toxic metabolites through reduction, hydrolysis, or oxidation [59,60]. These procedures require a reductive and oxidative enzyme that the fungus releases [61]. The second step is conjugation, which increases the nature of the intermediate products' water solubility [59]. Finally, peroxidases, oxygenases, and other enzymes convert DDT-intermediate metabolites into non-toxic compounds [62]. Lastly, benzaldehyde is produced when lignin-degrading enzymes split the molecule's ring structure [63], [64], [65], [66].

**Table 1:** Microbial species capable of degrading DDT.

**DDT degrader microbes**	**Microbial species**	**References**
Bacteria	*Ralstonia pickettii bacterium*	[72]
	*Staphylococcus hominis*	[73]
	*Pseudomonas aeruginosa*	[74]
	*Arthrobacter globiformis*	[75]
	*Kocuria rhizophila*	[76]
	*Staphylococcus equorum*	[76]
	*Staphylococcus cohnii*	[76]
	*Enterobacter cloacae*	[29]
Fungi	Gloeophyllum trabeum	[50]
	Trichoderma hamatum	[77]
	Pleurotus eryngii	[74]
	Rhizopus arrhizus	[78]
	Alternaria species	[78]
	Penicillium species	[70]
	Aspergillus niger	[79]
	Allescheriella species	[80]
	Phlebia species	[81]
	Paecilomyces species	[82]
	Fusarium oxysporum	[83]
	Phanerochaete chrysosporium	[84]
Algae	Chlamydomonas species	[85]
	Chlorella vulgaris	[86]
	Cylindrotheca species	[66]
	Euglena gracilis	[87]
	Scenedesmus obliquus	[88]
	Dunaliella salina	[43]
	Aulosira fertilissima	[88]
	Chlorococcum species	[88]
	Anabaena species	[45]
	Cladophora species	[89]
	Cladophora gracilis	[90]

The combined microbial remediation technology refers to the combination of two or more microorganism remediation methods to enhance the remediation of organic pollutants and improve the remediation efficiency. Fungi-bacteria interactions could lead to synergistic interaction effect. It can facilitate the degradation of DDT pollutants from diverse environmental matrices. The most broadly used combined microbial DDT remediation technology in DDT-contaminated environmental cleanup. Fungi and bacteria cometabolize in mixed microbial co-cultures, potentially accelerating and eliminating DDT's harmful effects and enhancing resistance to shifting or variable environmental conditions [67]. In addition, microalgae-bacteria-consortia approaches have synergistic effects for the microbial DDT remediation technique. These synergistic relationships have the potential to significantly enhance the biological DDT pollution treatment system, benefiting bacteria as well as microalgae [68,69]. It has been shown that a consortium of different fungi, in contrast to single strains, can degrade DDT pesticides more efficiently. Fungi-fungi remediation is the less-used combined bioremediation technology in DDT-contaminated soil and water ecosystems. It can take advantage of both different species of fungi. In some cases, the combined application of fungi and certain minerals has also proven more efficient for pesticide removal than the application of fungi or minerals alone [70,71].

## 3 Microbial DDT remediation mechanisms

**Fig. 1:**
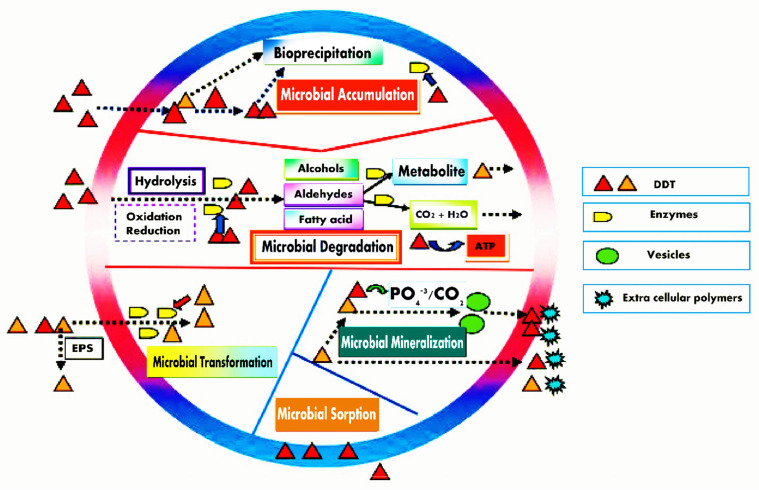
The mechanisms of microbial remediation used for degradation of DDT pesticides.

George M. Robinson used bacteria as a bioremediation technique to lessen the impact of oil spills for the first time in the 1960s. Microbial remediation mostly relies on microorganisms (micro-remediation) and microalgae (algal micro-remediation) to degrade pollutants. The DDT residue in soils and water has become an increasingly major environmental concern because of its widespread use in agriculture [91,92]. Microorganisms in DDT-contaminated soil and water use a variety of defense mechanisms to mitigate their toxicity [93]. An effective and promising method to clean up DDT-contaminated soils and water is microbial remediation, which uses microorganisms to detoxify DDT contaminants [63]. This section tries to discuss the currently recognized microbial remediation mechanisms of DDT pesticides in contaminated soils and water because the underlying mechanisms of microbial remediation of DDT-contaminated soil and water are not fully understood. Microbial remediation of DDT-pesticide contaminants can be grouped into five subclasses: microbial DDT sorption, microbial DDT accumulation, microbial DDT transformation, microbial DDT mineralization, and microbial DDT degradation (Fig. 1).

### 3.1 Microbial DDT sorption

DDT contaminants bind to microorganisms by a mechanism known as biosorption, which is independent of metabolism [94]. Some of this processes involved in microbial sorption include complexation, electrostatic interaction, ion exchange, physical and chemical adsorption, surface adsorption, diffusion, chelation, and microprecipitation [95]. Most of this process takes place in the cell wall [69]. The initial barrier preventing DDT contaminants from entering cells is the microbial cell wall, where they may be deposited on the surface or within the wall structure [93,85]. The mechanisms of microbial sorption allow hydrophobic substances to cross membranes and enter the organic matrix because of their lipophilic characteristics [96]. Through diffusion and chemical processes such as ion exchange, complexation, and others, contaminants like DDT and its residues can become adsorbed on cells. Additionally, functional groups (such as amine and hydroxyl groups) contribute to the microbial sorption of DDT contaminants [97].

### 3.2 Microbial DDT accumulation

Microbial DDT accumulation is the process by which DDT-pollutants pass through the cell membrane, enter the cytoplasm, and then go through the metabolic cycle of the cell [96,98]. The combination of physical, chemical, and biological mechanisms is known as accumulation. DDT-microbial accumulation happens when the concentration in the biosphere is significantly higher than in the surrounding area. As a result of microbial accumulation, environmental pollutants harm the microbes [99,100]. The lipophilic nature of DDT pesticides makes them more easily absorbed in cells due to the lipophilic components on the cell membrane [101]. Particulate, insoluble, and by-product pollutants build up in the cellular components of biological cells [100,101].

### 3.3 Microbial DDT transformation

Microbial DDT transformation is the conversion of DDT into another form in the body of microorganisms by enzymatic reactions [26]. Due to their changed physical and chemical properties, successful microbial remediation of DDT may lead to either a transformation into a low-water-soluble state or a water-soluble and low-hazardous state [26]. The enzymes or metabolites produced by microorganisms are mostly used in this process. For instance, fungus and bacteria can create biosurfactants that increase the bioavailability of DDT and enhance the effectiveness of micro-remediation [101,102]. In numerous instances, fungi and bacteria in microbial communities have shown the efficacy of DDT transformation [102,103].

### 3.4 Microbial DDT degradation

The microbial degradation of DDT involves a key mechanism called metabolism, in which microorganisms growing at the expense of a growth substrate are able to convert DDT without getting any nutrients or energy for growth [70,72,104]. Microbial degradation of DDT has been documented in different literatures. The majority of reports show that, in reducing conditions, DDT is reductively dechlorinated to DDD [29,71,105]. It has been shown that fungi and bacteria are capable of metabolizing DDT in this way, and the biodegradation pathways utilizing this method have been established. The strains of bacteria and fungi that degrade DDT have been identified as an alternate pathway for microbial attack in aerobic environments [18,29,53,71,106].

### 3.5 Microbial DDT mineralization

**Fig. 2:**
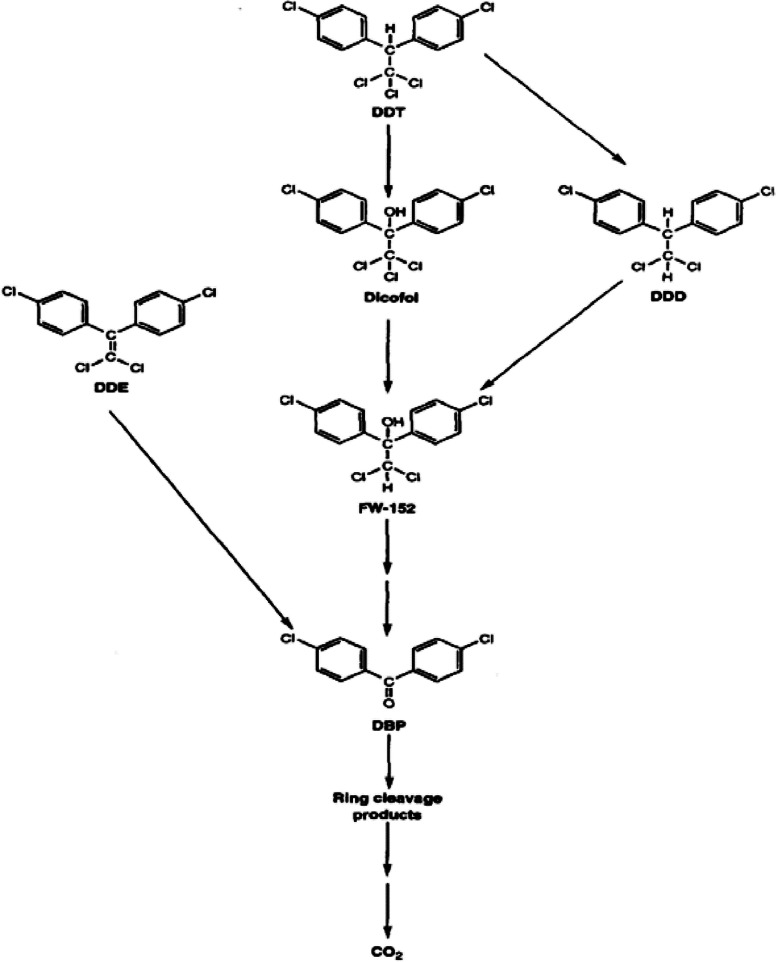
Microbial mineralization of DDT Proposed pathway for DDT and its residual degradation by Phanerochaete chrysosporium modified from [84].

Microbial DDT mineralization is the process by which DDT and its metabolites are converted into water and carbon dioxide in cells and some tissues under the influence or direction of biological organic matter (Fig. 2) [51,107]. Biologically induced mineralization and biologically regulated mineralization are two mechanisms of microbial DDT-mineralization.

**Fig. 3:**
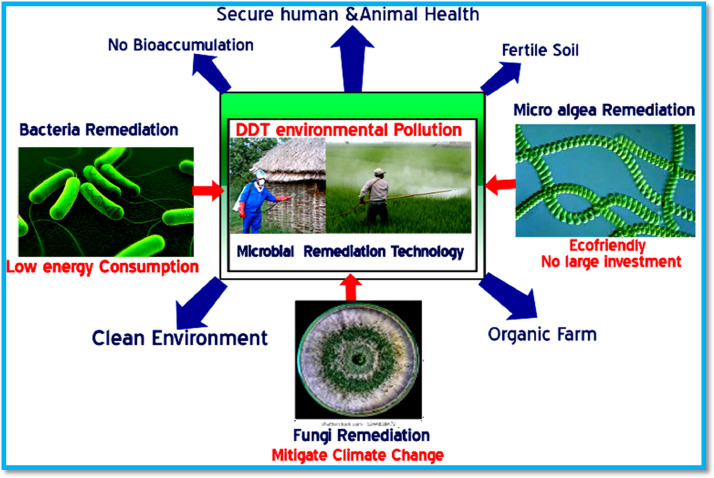
Microbial remediation of DDT from waste water and farm soil.

## 4 Microbial enzymes in DDT remediation

**Fig. 4:**
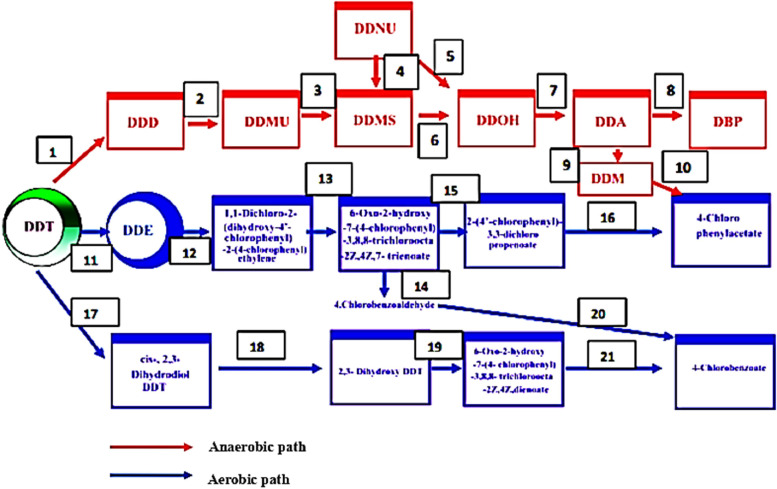
Mechanistic scheme of DDT enzymatic microbial degradation under anaerobic condition (red) path way DDT reductive dechlorinase (1); DDD dehydrochlorinase (2); DDMS dehydrogenase (3); DDNU hydratase (5); DDMS dehalogenase (6); DDA decarboxylase (8); DDA decarboxylase (9), DDE dehalogenase (22) and under aerobic conditions (blue) path way DDT dehydrochlorinase (11), 1,1-dichloro-2,2-bis(p-chlorophenyl) ethylene dioxygenase (13, 14), 6-oxo-2‑hydroxy 7-(4′-chlorophenyl) 3,8,8-trichloroocta-2Z,4Z,7- trienoate hydrolase (15), 4-chlorobenzaldehyde dehydrogenase (14), 4-chlorobenzoaldehyde dehydrogenase (20), DDT 2,3-dioxygenase (17), cis-2,3-dihydrodiol DDT dehydrogenase (18), and 2,3-dihydroxy DDT 1,2-dioxygenase (19) Factors Affecting Enzymatic Degradation of DDT.

The DDT pesticide degrading gene resides in an anti-catabolic plasmid and encodes the DDT degradation enzymes [108]. For instance, in many gram-negative soil bacteria that degrade DDT, the Lin gene encodes many enzymes, including hydrolase, dehydrogenase, and dehalogenase [108]. Microbial genes such as atz, ndo, psb, puh, tfd, tri, and trz encode different groups of enzymes involved in the degradation of DDT. It involves oxidoreductases (such as laccases, oxygenases, and peroxidases) and hydrolytic enzymes (such as cellulases, lipases, and proteases) [49]. Microbes release certain catalytic enzymes (including oxidoreductases, oxygenases, monooxygenases, dioxygenases, laccases, and peroxidases) to degrade complex pollutants such as DDT [49]. These enzymes play a role in the DDT microbial remediation process by breaking the chemical bonds and reducing the toxicity of the DDT pollutant (Fig. 4) [108].

### 4.1 Laccases enzymes

Since laccase enzymes are members of the multicopper oxidase family, they are also known as benzenediol dioxygen oxidoreductases or p-diphenol oxidases. They oxidize a variety of substrates while also reducing molecular oxygen to water during the microbial DDT mineralization process [109]. Insects, higher plants, bacteria, fungi, and other microbes all have laccase enzymes in different quantities for DDT biodegradation mechanisms [110]. In higher plants and fungi, laccases are typically found. However, it has recently been shown that some bacterial species, including S. lavendulae, Marinomonas mediterranea, Pseudomonas sp., and Bacillus sp., also secrete laccases [111,112]. The basidiomycetes that produce laccase enzymes are Phanerochaete chrysosporium, Theiophora terrestris, Lenzites betulina, Phlebia radiata, Pleurotus ostreatus, and Trametes versicolor [112]. These fungi's laccase enzymes break down the complex polyphenolic lignin of DDT and its residue. DDT could be converted into a harmless or nontoxic byproduct using ligninolytic enzymes that can be produced by microorganisms [109].

Furthermore, bacterial laccases can be more active and stable under high-temperature, high-pH, and high-alkaline conditions than fungal laccases [113]. These enzymes have been gaining attention for biotechnological applications in recent years due to their extracellular and inducible nature, lack of cofactor requirements, and low specificity [[114], [115]]. The addition of numerous chemicals, including copper, dyes, and other substances, can increase laccase synthesis [116]. These enzymes allow the breakdown of DDT and its metabolites because of their low substrate specificity. Unlike peroxidases, which use hydrogen peroxide as an electron acceptor, a laccase-catalyzed process utilizes oxygen from the atmosphere [115]. DDT and its main metabolite, dichlorodiphenyldichloroethylene (DDE), are ubiquitous environmental contaminants [117]. The white-rot fungus laccase, which Polyporus produces, was used to remove DDT from soil [118]. Their findings demonstrated that laccase can efficiently break down DDT after only 25 days of incubation [118].

### 4.2 Oxidoreductases

Oxidase enzymes help certain bacterial species use an oxidative coupling process. It detoxifies toxic and resistant chemical substances like DDT and converts them into less harmful ones [119]. Fungi use a biochemical mechanism known as the oxidoreductase biocatalyst to absorb energy from the body. This reaction breaks chemical bonds to help transport electrons from the reduced organic component to an alternative chemical molecule [120]. These oxidation–reduction processes ultimately result in the contaminants being changed into less hazardous molecules [120].

Most microbial species have the capacity to eradicate chlorinated aromatic organic compounds, such as DDT, from polluted areas. It is primarily the result of extracellular oxidase enzymes. These enzymes are found in the adjacent environment and are separated from the fungal body (mycelium). Examples of these enzymes are manganese, lignin, laccase, and peroxidase. Furthermore, as compared to bacteria, fungi could efficiently colonize the soil pollution because of their filamentous structure [119].

### 4.3 Oxygenases

Oxygenase enzymes belong to the oxidoreductase group. Based on the number of oxygen atoms, oxygenases are classified into two groups: monooxygenases and dioxygenases [121]. They are very important in the metabolic process of organic compounds and enhance water solubility [122]. These enzymes have a wide range of substrates and are effective against a wide range of compounds, such as DDT compounds [108]. Oxygenase enzymes break the aromatic rings through the overview of oxygen atoms into organic molecules. It is a very important enzyme in the DDT remediation process [123]. A particular oxygenase enzyme aids in the breakdown of DDT contaminants. Oxygenases also dehalogenate halogenated ethylenes, ethanes, and methanes [119].

### 4.4 Monooxygenases

Monooxygenases are stereo-selective and region-selective enzymes that perform DDT microbial remediation processes as biocatalysts [124]. Most of the monooxygenase enzymes consist of cofactors, but some monooxygenases do not need cofactors for their function. These enzymes simply need oxygen for their achievements [125]. It plays a role in the dehalogenation, desulfurization, denitrification, hydroxylation, ammonification, biodegradation, and biotransformation of DDT compounds [126]. It al5so participates in the decomposition of hydrocarbons, viz., alkanes, cycloalkanes, methanes, alkenes, haloalkenes, aromatics, and ethers [126]. Monooxygenases carry out the oxidation of dehalogenation reactions and dehydrochlorination in oxygen-rich environments. However, in oxygen-less environments, the reduction of dechlorination reactions is carried out. The oxidation of the substrate during dehalogenation results in labile products, which are then chemically decomposed [127].

## 5 Microbial DDT remediation strategies

DDT remediation strategies for detoxification of DDT mainly depend on physical, chemical, and biological approaches. These approaches can be used alone or in combinations [128,129]. Cleaning DDT from the environment can be carried out adequately, both ex situ and in situ, according to the need or not to remove contaminated soil [130].

### 5.1 ***In situ*** microbial **DDT** remediation **strategies**

In-situ microbial remediation techniques are preferable over ex-situ techniques for the treatment of DDT-contaminated water and soil ecosystems due to their low cost**,** minimal maintenance, environmental friendliness, and sustainability [130]. In situ microbial DDT remediation can be done in three different ways: bio-attenuation, bio-stimulation, and bioaugmentation [131,130].

#### 5.1.1 Bio-attenuation approach

DDT toxic contaminants are converted into less toxic or non-toxic forms by microbial degradation, with their interaction by naturally existing chemicals, and their sorption on geologic media can facilitate bio-attenuation. This mechanism is efficient and cost-effective, but it also takes a long time, depending on the conditions of the DDT-contaminated location. It may not require a complete clearing up of DDT [24,132].

#### 5.1.2 Biostimulation approach

The method of changing the environment to promote the growth of microorganisms capable of DDT remediation is known as biostimulation. This can be achieved by supplementing insufficient amounts of electron acceptors and limiting nutrients such as carbon, nitrogen, phosphorus, or oxygen, which restrict microbial activity. The detoxification potential for DDT pesticides under liquid media conditions has numerous factors that affect microbial growth. These factors are nutrients, pH, temperature, moisture, oxygen, soil characteristics, and the presence of DDT pollutants, which might prevent DDT from biodegrading in soil and water. The primary advantage of biostimulation is that microbial remediation of DDT will be carried out by indigenous microbes that have already existed, are widely dispersed, and are well-suited to the subterranean environment [133]. Both bioaugmentation and microbial-remediation techniques can work together to enhance the ability of microbes to degrade DDT compounds and optimize the efficiency of the process [134].

#### 5.1.3 Bioaugmentation approach

Bioaugmentation is a green technology that is known as an enhancement of microbial DDT degradation capability by applying it to specific contaminated areas [48]. Specific kinds of microbial groups can enhance DDT breakdown significantly. This technology is mostly applied in areas where inhabitant microorganisms do not have a significant degradation capability of DDT [135]. Bioaugmentation is mainly enhancing the catabolic potential of the microorganisms for the recovery of contaminating agents (DDT). To accomplish this objective, the inoculation of desired microbial species can be carried out. Besides these, genetically modified and engineered organisms (GMOs) are extremely suitable for DDT degradation and enhanced bioaugmentation processing. Several microbial growth variables affect bioaugmentation. An appropriate strain selection is a very important factor. Therefore, planning inoculum development should also be taken into consideration [91]. The strains should have a high potential for DDT degradation, and the selected strain could colonize the DDT-contaminated area within a short period of time [136]. The development of strains has a higher capacity to survive under high DDT concentrations [71,75,137]. Furthermore, the strain can efficiently survive under a wide range of harsh and suitable environmental conditions [75]. For DDT pesticide degradation, new microbial species should be investigated that have better and faster degradation capacity in contaminated soil environments [138]. Collecting microorganisms from DDT-contaminated ecosystems is a good source for bioaugmentation [139**].** It is crucial to determine how DDT pesticide detoxifying capacity in liquid media settings contributes more effectively to the bioaugmentation of microbial consortiums [140].

### 5.2 *Ex-situ* microbial DDT remediation strategies

*Ex-situ* microbial DDT remediation of water and soil demands the laborious, expensive, and time-consuming removal of DDT-contaminated water and soil from the site to be treated elsewhere [130,141]. Bioreactors can also be categorized as an *ex-situ* microbial remediation technique [141]. The use of bioreactors in microbial remediation techniques provides a number of advantages. These are better control and management of factors like temperature, pH, agitation, and aeration, as well as the option of enhancing the process by incorporating different optimization strategies [142]. To remediate the partial or complete elimination of DDT and transform it into elemental components, microbes utilize DDT as a carbon source, which mostly depends on the presence of DDT residues and microbes in the soil [143]. The retention of DDT residues with colloidal particles can significantly make them unavailable to microbes, which will affect their degradation. More than 10,000 fungal colonies are present in 1 g of bulk soil, approximately. These fungal communities are highly involved in DDT residue degradation in the ecosystem over time [144].

Fungal species used in microbial remediation techniques are obtained from bulk of soil and decaying wood. Thirteen fungal species were investigated in the removal process of DDT residues, which included Ascomycota (7 species), Basidiomycota (4 species), and Glomeromycota (2 species) [48]. DDT is very persistent in the soil environment as well as in the water ecosystem, but many fungal, bacterial, and micro-algae species have been identified as involved in the breakdown and detoxification of DDT (Fig. 3) [48,135]. The effectiveness of the DDT remediation process depends on environmental factors such as temperature, moisture content, organic matter content, redox status, and pH [48]. The enzymatic activity of microbes to degrade DDT pesticide depends on pH, and most enzymes work efficiently at pH ranges of 6.5 and 7.5. A decrease in pH can affect bioavailability and remediation rates significantly [48]. The abundance of organic matter provides many nutrients for microbial food, which will increase their working efficiency for higher degradation of DDT contaminants [48].

Microbes release some chemicals that can increase DDT degradation potential [137,145]. These chemicals may also stimulate the extracellular degradation of DDT contaminants [24]. This will also increase the DDT remediation process and efficiency because it does not require energy to transport the contaminants in the fungal body [132]. Successful microbial remediation with the use of free enzymes is also investigated [59].

## 6 Trends in enzymatic DDT degradation methods

### 6.1 Enzyme immobilization

Microbial enzymes that have been immobilized are used in a wide range of scientific and environmental cleaning procedures to remove persistent organic pollutants like DDT [108]. Enzymes are immobilized into strong, stable supports to maintain high stability in terms of pH, temperature, packaging, reuse, and separation [146]. A current study reports that horseradish peroxidase (HRP) was cross-linked onto calcium-alginate beads using glutaraldehyde as a cross-linking agent to help with the covalent immobilization of HRP, which is necessary for the breakdown of DDT [59].

### 6.2 Genetic engineering approach

Enzyme utilization is restricted in industrial-scale environmental applications due to the economics of enzyme processing [147]. The researchers used a genetic engineering method to increase the yield of enzyme production to remediate environmental pollutants such as DDT [148]. This can be achieved using one of two methods: genetic experimentation to pick natural forms or mutagenesis of a gene in vitro and expression in a host cell. Laccase enzymes are predominantly employed for genetic manipulation. The laccase gene from Aspergillus niger cDNA was used for cloning for DDT degradation purposes [149].

## 7 Main factors affecting microbial DDT remediation

In order to sustain the high bioavailability of microorganisms in soil and water for the complete DDT remediation capability of microorganisms, a specific combination of biotic and abiotic variables is required [97]. A number of variables affect the complete microbial DDT remediation process. The same variables that have an impact on the microbial remediation of contaminated soils and waters also have an effect on the microbial remediation of DDT contaminated soil and waters. Abiotic and biotic variables are most likely to have an impact on the microbial remediation of DDT contaminated soil and water [150].

### 7.1 Abiotic factors

The microbial remediation of DDT could be significantly affected by abiotic factors such as pH, temperature, soil type, water, soil moisture, cation exchange capacity (CEC), redox potential (Eh), nutrient sources, exudate quantity, rhizosphere environment, and the composition and biochemical processes. These factors can influence the microbial growth in DDT-contaminated soil and water environment [143,150]. DDT becomes more soluble at higher temperatures, which increases the pesticide's bioavailability [143,148,151]. Furthermore, insufficient soil moisture can make the microbial DDT remediation process more difficult. Low soil moisture content hinders the growth and metabolism of microorganisms, and high values reduce soil aeration [97]. Additionally, the type of soil has a significant impact on the bioavailability of DDT remediation in the soil [152]. The texture of soil particles has a direct impact on the availability of potential DDT-degrading microbes [153]. Loam and sand have the maximum DDT availability, while fine-textured clay soils and clay loam have the lowest availability [154]. Clay content, oxygen concentration, salinity, minerals, and the availability of nutrients can all have an impact on microbial DDT remediation [155]. Organic matter content and soil types can influence how microbes react to DDT pollutants [60].

The enzymatic dosage has played a crucial role in the microbial DDT remediation process [26]. Typically, DDT degradation efficiency rises to a certain point with elevated enzyme concentrations [59]. Consequently, DDT remains persistent due to the limited concentration of microbial enzymes available both in the batch and environmental system [59]. Research by Bakshi et al. [128] examined the ability of MnP from *Bacillus velezensis's* to decompose DDT. The batch experiments were conducted with varying inoculum ranges from 1 % to 11 % (w/v) of the MnP enzyme. An optimal inoculum of 7 % (w/v) was observed to be best for obtaining higher DDT degradation efficiency [156,157]. Agitation is also considered a crucial factor for DDT-microbial remediation, as it is primarily responsible for heat, substrate, and oxygen transfer in the process. A higher speed of agitation results in enhanced biodegradation efficiency owing to better oxygen availability in aerobic processes. Speeds of 200 rpm/min resulted in increased DDT-degradation potential [59].

#### 7.1.1 pH

pH has an effect on microbial DDT remediation because different microbial species have different optimal pH ranges. Soil pH can have a variety of effects on microbial DDT remediation [94]. At extreme pH, some microbial degradation processes are inhibited. Some still have the potential to remedy DDT pollution in suboptimal conditions [158]. The optimal pH varies for different enzymes derived from different microorganisms. A pH value outside the optimal range results in lower enzyme activity. Most of the enzymes work in a neutral pH range. However, some enzymes, such as alkaline proteases, work at pH levels above 8.0. It was studied how lignocellulolytic enzymes from white-rot fungi, namely manganese peroxidases (MnP) and lignin peroxidases, broke down DDT through microbial degradation. For crude enzymes, the optimum pH for microbial degradation has been found at pH 5 [61].

#### 7.1.2 Temperature

Temperature has an impact on the chemical composition as well as the physical properties of DDT contaminants [157]. Temperature has a direct impact on how DDT pesticide adheres to microorganisms, soil, and particles during the adsorption and desorption processes [159]. With rising temperatures, DDT's adsorption capacity and intensity will increase [160]. The DDT-breakdown process by microorganisms benefits from an increase in temperature within a suitable range [161]. Temperature affects the activity of both intracellular and extracellular enzymes of microbes in DDT degradation mechanisms [75]. Microbial enzymes have a variety of optimal enzymes for DDT degradation [162]. Because enzymes are proteins, they are especially susceptible to high temperatures, which may cause the protein to become denatured or alter its characteristics [75]. It was discovered that Pseudoxanthomonas sp. that breaks down DDT, thrives best in temperatures between 20 and 37 °C. This is because higher temperatures that cause the laccase enzymes to either become inactive or cleave [163]. The immobilized enzymes produced extracellularly by white-rot fungi demonstrated remarkable activity and stability across a wide temperature range (20 to 55 °C). This biocatalyst effectively degraded p,p'-DDT, completely eliminating it within a 12-hour incubation period at 30 °C [61].

#### 7.1.3 DDT effects on microbial remediation

DDT pesticides can directly affect organisms as well as microbial remediation procedures [26]. Some microbes can thrive in DDT-contaminated areas, despite the potential toxicity, due to resistance or tolerance mechanisms [71,155,164]. However, it has still variable degrees of effect on its biomass, physiological morphology, growth, microbial activity, and mycelia colonization [71,155]. DDT pesticide exposure may change the physiological and biochemical behavior of soil and water microorganisms [155,165]. DDT-insecticides interfere with some crucial processes and inhibit a number of enzymes, disrupting the mechanism that microbes use to promote their growth [165,166]. DDT has an impact on the number and diversity of microorganisms, biomass production, and shape of microbes [71,166]. Notably, while DDT is hazardous to some organisms, some microorganisms can utilize DDT pesticides in low concentrations as a source of nutrients or energy to reproduce and promote development throughout the microbial remediation process [71,110,167]. Occasionally, over time, microbial populations can develop tolerance or resistance to DDT, respond to normal conditions, or even increase DDT concentration. [71,168,169].

### 7.2 Biotic factors

The ability of microbial DDT remediation in soil and water is determined by biotic factors such as inoculum density, survival, colonization, competitiveness, microbial activity, physical diffusion capacities, etc. [92,169]. The success of DDT bioremediation depends on the species' ability to survive in these stressful environments. A major factor limiting the effectiveness of microbial DDT remediation and strongly influencing the success or failure of microbial remediation is bioavailability. These factors influenced biological activity like species types, microbial biomass, symbiotic species, and bioavailability [94,150].

## 8 Future perspectives on microbial industrial applicability

Microbial-based DDT remediation techniques have been proven to be an effective, low-energy, environmentally friendly, and sustainable way to remove DDT contaminants in soil and wastewater. For wide-scale industrial applications, there are a few issues that must be resolved. Most microbial DDT remediation research is focused on treating wastewater and soil in batches, but continuous flow methods for large-scale industrial applications ought to be investigated. These systems that impact microbial growth patterns are additionally controlled by changing environmental conditions. Further study should be done to increase the transgenic microorganisms' survivability when released into the environment, as their detoxification and breakdown of DDT contaminants have been greatly enhanced. For effective and improved microbial growth for DDT remediation, a careful examination of the microbial DDT degradation operating parameters, microbial DDT remediation process mechanisms, and environmental condition is necessary. A complex harvesting technique, an early stage of downstream processing, and a high fertilizer requirement for algal growth are further barriers to microalgae-based DDT degradation.

## 9 Conclusion

The amount of DDT insecticide released into the environment has greatly increased due to anthropogenic activity. It can potentially have a negative impact on human health and endanger natural ecosystems. It has been demonstrated that microbial DDT remediation is an effective method for cleaning up DDT-contaminated soils and water. Microbial enzymes and DDT cleanup can be a useful, economical, and environmentally friendly method of eliminating DDT. DDT residue and its hazardous chemicals have been biotransformed into harmless forms using microbial enzymes. Microbes and their enzymes efficiently degrade the high concentration of DDT by utilizing it as a carbon source. Abiotic and biotic parameters, such as pH, temperature, microbial type, incubation age, and contaminant availability. These parameters influence the possible microbial DDT remediation method and its enzymatic function in contaminated soil and water. It must be taken into consideration for effective DDT degradation. Microbial inoculum development processing and genetic modification techniques have made it possible to develop microbes with improved DDT remediation capabilities and enzymes with better physiological conditions that allow processes to be carried out in stressful environments. For microbial DDT remediation mechanisms, numerous in vitro investigations were conducted. The parameters of the experimental design were chosen to best suit the climate, weather, and DDT pollution levels. Instead of using pot experiments and lab settings, more study should be needed to confirm the DDT bioremediation efficacy in contaminated field settings. The field applications ought to be conducted in various places. Potential novel species must be screened, either naturally or artificially, for practical applications. Enzyme systems that produce the best yields and efficiency with the least amount of water, energy, and nutrients need to be developed. Even though genetic engineering methods produce more enzymes with greater efficiency, more investigation is required to tackle the genetic engineering method's financial challenge. In the same way, researchers must also find solutions to the issues surrounding enzyme immobilization, another advanced strategy that improves enzyme effectiveness for DDT breakdown.

## CRediT authorship contribution statement

**Girma Ebsa:** Writing – original draft. **Birhanu Gizaw:** Writing – review & editing, Writing – original draft. **Mesele Admassie:** Writing – review & editing. **Tizazu Degu:** Writing – review & editing, Writing – original draft. **Tesfaye Alemu:** Writing – review & editing.

## Declaration of competing interest

The authors declared that they do not have any known competing financial interests or personal relationships that could have appeared to influence the work reported in this paper.

## Data Availability

The data that has been used is confidential.
